# Methylation-based reclassification and risk stratification of skull-base chordomas

**DOI:** 10.3389/fonc.2022.960005

**Published:** 2022-11-11

**Authors:** Xulei Huo, Tengxian Guo, Ke Wang, Bohan Yao, Da Li, Huan Li, Wei Chen, Liang Wang, Zhen Wu

**Affiliations:** ^1^ Department of Neurosurgery, Beijing Tiantan Hospital, Capital Medical University, Beijing, China; ^2^ Center of Brain Tumor, Beijing Institute for Brain Disorders, Beijing, China; ^3^ Department of Neurosurgery, Beijing Fengtai Hospital, Beijing, China; ^4^ Beijing Advanced Innovation Centre for Biomedical Engineering, Key Laboratory for Biomechanics and Mechanobiology of Ministry of Education, School of Biological Science and Medical Engineering, Beihang University, Beijing, China; ^5^ Beijing Neurosurgical Institute, Capital Medical University, Beijing, China

**Keywords:** DNA methylation, prognostic biomarker, infiltration immune level, stemness indices, copy number variation

## Abstract

**Background:**

Skull-base chordomas are rare malignant bone cancers originating from the remnant of the notochord. Survival is variable, and clinical or molecular factors cannot reliably predict their outcomes. This study therefore identified epigenetic subtypes that defined new chordoma epigenetic profiles and their corresponding characteristics.

**Methods:**

Methylation profiles of 46 chordoma-resected neoplasms between 2008 and 2014, along with clinical information, were collected. K-means consensus clustering and principal component analysis were used to identify and validate the clusters. Single-sample gene set enrichment analysis, methylCIBERSORT algorithm, and copy number analysis were used to identify the characteristics of the clusters.

**Results:**

Unsupervised clustering analysis confirmed two clusters with a progression-free survival difference. Gene set enrichment analysis indicated that the early and late estrogen response pathways and the hypoxia pathway were activated whereas the inflammatory and interferon gamma responses were suppressed. Forty-six potential therapeutic targets corresponding to differentially methylated sites were identified from chordoma patients. Subgroups with a worse outcome were characterized by low immune cell infiltration, higher tumor purity, and higher stemness indices. Moreover, copy number amplifications mostly occurred in cluster 1 tumors and the high-risk group. Additionally, the presence of a CCNE1 deletion was exclusively found in the group of chordoma patients with better outcome, whereas RB1 and CDKN2A/2B deletions were mainly found in the group of chordoma patients with worse outcome.

**Conclusions:**

Chordoma prognostic epigenetic subtypes were identified, and their corresponding characteristics were found to be variable.

## Introduction

Skull-base chordomas are rare malignant bone tumors that originate from the remnant of the notochord (1) and can develop on the sacrococcygeal area (2). These chordomas have infiltration characteristics, which make them difficult to safely remove, thus leading to high recurrence and malignant progression. Additionally, the recurrence of chordomas is very high in skull-base cases, although they grow slowly and can be satisfactorily resected (3). Moreover, clinical progression is more malignant with the occurrence of a recurrence status. However, the treatment and the identities of molecular biomarkers are still poorly characterized.

Accurate subtype identification and prognostic stratification are helpful for treatment and for understanding potential mechanisms contributing to the progression of cancers. DNA methylation is often associated with gene silencing because it inhibits the interaction of chromatin with DNA-binding proteins or transcription factors required for gene expression (4). In addition, DNA methylation profiling has been proven to be a useful signature for subgroup samples and for redefinition of risk scores (5–10). Chordoma methylation profiles have been reported in some studies (11–13), especially in a recent study reporting that chordomas could be divided by epigenetic changes and could be used as prognostic factors for clinical outcomes (14). CA2 (carbonic anhydrase II) has been identified in switched compartments, cell-specific boundaries, and loops (15) of chordomas. Inhibition of histone H3K27 demethylases inactivates brachyury (TBXT) and promotes chordoma cell death (16). Cancer-specific, differentially methylated loci are involved in various networks, including cancer, nervous system development and function, cell death and survival, cellular growth, cellular development, and proliferation (12). In this study, we used 46 skull-base chordoma samples to identify methylation-based prognostic subtypes with the help of unsupervised hierarchical clustering and copy number variation profiles. Signatures that could predict skull-base chordoma outcomes based on methylation-based prognostic subtypes were then identified and validated.

## Materials and methods

### Patient cohort

A total of 46 patients were enrolled in this study. Patients were treated with surgery between 2008 and 2014. Reviewers blinded to the original diagnoses conducted histological assays using formalin-fixed, paraffin-embedded hematoxylin and eosin sections, and selected tumor tissues to obtain DNA methylation profiles. The Ethics Committee of Beijing Tiantan Hospital approved this study, and all patients signed written informed consent forms for the study. Clinical information including age, sex, therapeutic modalities, and follow-ups was recorded. The extent of resection was identified based on surgical record information and postoperative magnetic resonance imaging (MRI) within 1 month of treatment. The definition of gross total resection involved no evidence of residual intraoperative neoplasm using postoperative MRI. Conversely, the definition of subtotal resection was any evidence of residual intraoperative neoplasm using postoperative MRI. Disease-specific survival was defined as the interval between the time of surgery and death or tumor progression.

### Methylation profiling of chordoma tissues

Total DNA was extracted from fresh frozen tissues. Approximately 400 ng bisulfite-converted DNA was then profiled using an Infinium Methylation 450K BeadChip array (Illumina, San Diego, CA, USA) according to the manufacturer’s protocol. The.idat files, including our cohort and external data sets, were processed using the minfi package, and the data were normalized using the preprocessIllumina function. CpG sites involving low-quality data (one sample CpG detection with *p* > 0.01), those on the X and Y chromosomes, and the sites that overlapped with single-nucleotide polymorphisms were removed. The quality of sample controls was then assessed using *p*< 0.05 as the level of significance. Beta values were obtained for downstream analyses.

### Identification and validation of prognostic clusters

The K-values of two samples and optimal clustering parameters (10,000 CpG sites) were confirmed by silhouette scores and consensus cumulative distribution functions (17). K-means consensus clustering was performed with the optimal clustering parameters across all samples, with the results visualized using a heat map with dendrograms. Principal component analysis (PCA) was performed to define the above clusters, followed by visualization. The two clusters were compared using Kaplan–Meier plots with disease-free survival (DFS). Univariate and multivariate Cox regression analyses incorporating methylation-based clusters and clinical features were used to evaluate the independent factors predicting DFS.

### Differential methylation between prognostic subtypes

CpG sites were annotated using standard Illumina Infinium Methylation 450K BeadChip criteria. RefSeq genome annotations (hg19) were used to identify the exon regions and transcription start site coordinates. Regions at 2 kb upstream and downstream of the transcription start sites were defined as the promoter region. Only overlapping gene promoters were chosen after the CpG site was mapped to the genome. The DMP-CHAMP pipeline was assessed to identify the differential methylation sites between the two clusters and visualized using a volcano plot of probes that were selected based on the mean β value difference; Δβ > 0.15 and *p*< 0.05 were considered differentially methylated (18) (Benjamini and Hochberg *p*-value). Gene set enrichment analysis (GSEA) (19) was performed using differentially methylated gene data with the clusterProfiler package (20) (*p*< 0.05 of the false discovery rate). The hallmark gene sets (h.all.v7.4.symbols.gmt), which were downloaded from the Molecular Signatures Database, were used in the analysis. To further identify potential drug targets corresponding to the methylation sites in chordomas, the Open Targets database was used to screen the drug targets of chordomas (accessed in May 2022). The Open Targets database is a powerful data integration platform for potential identification and validation of diseases (21). To improve prediction accuracy, overlap of predicted genes from the Open Targets database and differential genes between cluster 1 and cluster 2 were used to represent the final results of predicted target genes.

### Identification and validation of prognostic signatures

First, a univariate Cox regression analysis was conducted to determine the relationship between the above differential methylation sites and DFS. Candidate methylated sites were defined as methylated sites having a significant statistical difference (*p*< 0.01). Using the glmnet package, least absolute shrinkage and selection operator (LASSO) analyses were then conducted to reduce the candidate methylated sites (22). Finally, a stepwise multivariate regression analysis was conducted to select the final methylated sites for a predictive model that had the lowest Akaike information value. Risk scores were then calculated using the methylated sites from the predictive model. The specific formula is as follows:


$$\text{risk score}=\sum^{}\;({\text{coefficient }}_{n}\times \text{ β value of site }1_{n}\text{) }$$

Based on the largest difference value between the true positive and false positive as the cutoff values, the patients were divided into high-risk and low-risk groups. Similarly, a Kaplan–Meier plot was used to compare the DFS between the two groups. In addition, using the timeROC package, the area under the curve (AUC) was calculated for 3, 5, and 10 years to assess the discriminability of the model (23).

### Single-sample gene set enrichment analysis and tumor microenvironment analysis

Single-sample GSEA (ssGSEA) was conducted to assess the immune infiltration level of 28 different immune cell types. The marker gene set of 28 different immune cell types was obtained from a published study (24), including innate immune cells (activated dendritic cells, CD56 bright natural killer cells, CD56 dim natural killer cells, eosinophils, immature dendritic cells, macrophages, mast cells, myeloid-derived suppressor cells, monocytes, natural killer cells, natural killer T cells, neutrophils, and plasmacytoid dendritic cells) and adaptive immune cells (activated B cells, activated CD4 T cells, activated CD8 T cells, central memory CD4 T cells, central memory CD8 T cells, effector memory CD4 T cells, effector memory CD8 T cells, gamma delta T cells, immature B cells, memory B cells, regulatory T cells, T follicular helper cells, Type 1 T helper cells, Type 17 T helper cells, and Type 2 T helper cells). As in our previous study (25), the ssGSEA algorithm transformed the marker gene expressions into infiltration levels of immune cells.

The composition of the tissue microenvironment was determined from DNA methylation data using the methylCIBERSORT package, which used the support vector regression adopted in CIBERSORT (26, 27). The methylCIBERSORT algorithm deconvoluted the samples into the fraction of estimated cancer cells, neutrophils, B lymphocytes, natural killer cells, cytotoxic T lymphocytes, monocytes/macrophages, regulatory T lymphocytes, effector T lymphocytes, endothelial cells, fibroblasts, and eosinophils. Additionally, the fraction of stromal cells, the fraction of immune cells, and the tumor purity in tumor tissues were determined using the ESTIMATE algorithm. This algorithm analyzed the corresponding gene expression signatures of immune and stromal cells to calculate immune and stromal scores that determined the infiltration level of tumor microenvironment cells (28).

### Immunohistochemistry validation

Immunostaining analysis was used to assess the CD8 (ab32620; Abcam, Cambridge, UK), CD56 (ab133345; Abcam), Treg (72338; Cell Signaling Technology, Danvers, MA, USA), and fibroblast (ab207178; Abcam) expression levels following the manufacturers’ protocols using formalin-fixed, paraffin-embedded tissue slides. The stained slides were independently evaluated by two experienced pathologists who were blinded to the identities of the samples. Discrepancies between the two observers were resolved by consensus.

### DNA methylation–based stemness indices

Stemness indices in the tumor were correlated with radiotherapy resistance, chemotherapy resistance, and the outcomes of patients. In 2018, pluripotent stem cell samples from the Progenitor Cell Biology Consortium data set were used to build a model that could predict stemness indices with the DNA methylation data using the OCLR algorithm (29, 30). The model to obtain stemness indices was found on https://bioinformaticsfmrp.github.io/PanCanStem_Web/. We used the stemness index model to score the stemness indices of our cohort and to transform the results in the [0, 1] range using a linear transformation that subtracted the minimum data and divided by the maximum data.

### Copy number alterations

Copy number alterations were obtained from normalized raw methylation data using the conumee package (Bioconductor). Amplification or deletions were defined with a log_2_ copy number ratio of >0.3 or a log_2_ copy number ratio of<−0.3 (31). Copy number alteration analyses were then identified in the external chordoma data sets, including 46 DNA methylation data from GSE14068629 (32).

### Statistical analysis

All statistical analyses were performed with R, version 4.1.2 (The R Foundation for Statistical Computing, Vienna, Austria), and a value of *p*< 0.05 was considered statistically significant. The Kaplan–Meier method was performed and compared using the log-rank test. Data are presented as the mean ± standard deviation. Fisher’s exact test was used to compare sex, extent of surgery, and adjuvant radiotherapy between groups. The age, disease-free time, immune scores, and stemness index difference between groups were analyzed using an independent samples *t*-test. Prism 9.0 software (GraphPad, San Diego, CA, USA) was used to plot the results (^***^ *p*< 0.001, ^**^ *p*< 0.01, ^*^p< 0.05).

## Results

### Identification of DNA methylation–based chordoma subtypes

**Figure 1:**
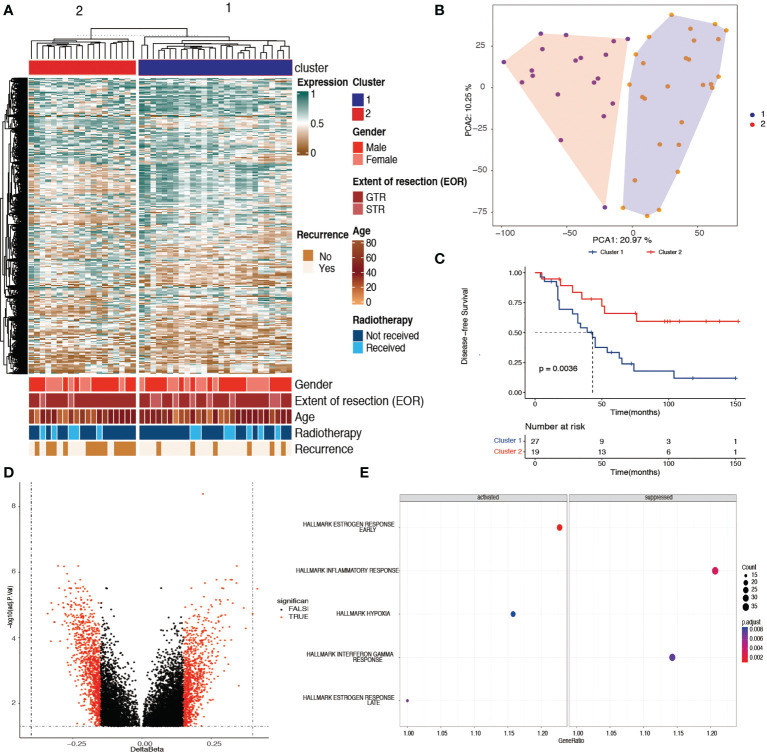
Identification of two prognostic chordoma subtypes with tumor DNA methylation signatures. **(A)** Unsupervised learning cluster identified two skull-base subtypes in the 46 DNA methylation samples with the most variable 10,000 probes. Color annotations display the distribution of clinical features (sex, age, extent of surgery, adjuvant radiotherapy, and the recurrence status). **(B)** Principle component analysis results of the cohort samples showed that the samples within cluster 1 were well separated from the samples within cluster 2, with the top 10,000 most variable methylated probes. **(C)** Kaplan–Meier plot showing cluster 1 had a worse disease-specific survival time when compared with cluster 2 (*p*< 0.01). **(D)** Volcano plot showing 1,496 probes with differentially methylated promoters between cluster 1 and cluster 2. **(E)** Gene set enrichment analysis revealing the activated and suppressed pathways within hallmark gene sets in cluster 1 when compared with cluster 2. The early and late estrogen response pathways and the hypoxia pathway were activated, whereas the inflammatory and interferon gamma responses were suppressed in cluster 1 (adjusted *p*< 0.05).

Unsupervised learning clustering of the 46 tumor samples using the top 10,000 most variably methylated CpG sites identified two clusters, with the corresponding clinical information shown in **Figure 1A**. To confirm the two clusters, PCA was used to further compare the DNA methylation profiles between the two clusters, which showed that the clusters had a clear distinction (**Figure 1B**). PCA results revealed that the samples within cluster 1 were well separated from the samples in cluster 2. The corresponding clinical information of the two clusters is shown in **Table 1** and **Supplementary Table S1**.

**Table 1:** Clinical characteristics of skull-base chordomas.

Characteristics	Combined cohort	Cluster 1 (n = 27)	Cluster 2 (n = 19)	*p*-value
Gender (male vs. female)	60.9% vs. 39.1%	59.2% vs. 40.7%	63.2% vs. 36.8%	1.00
Age (mean ± SD)	36.8 ± 14.2	37.1 ± 13.8	36.4 ± 15.1	0.88
Extent of surgery (gross total vs. subtotal)	78.3% vs. 21.7%	70.4% vs. 29.6%	89.5% vs. 10.5%	0.24
Adjuvant radiotherapy	73.9% vs. 26.1%	74.1% vs. 25.9%	73.7% vs. 26.3%	1.00
Disease-free survival (months) (mean ± SD)	58.1 ± 41.9	45.3 ± 35.1	76.3 ± 44.9	0.01

### Characterization of the two clusters

The baseline clinical information of the 46 chordoma samples is listed in **Table 1**. There was no difference in sex, age, extent of surgery, and adjuvant radiotherapy between the two clusters. In addition, cluster 2 had a significantly better DFS than cluster 1 (**Figure 1C**, log-rank test, *p* = 0.0036). The mean survival time was 45.3 months in cluster 1, whereas in cluster 2, it was 76.3 months.

**Table 2** shows the results of univariate and multivariable regression analyses combining the clinical information and the methylation-based clusters. Using univariate analysis, the cluster was a statistically significant independent prognostic factor [cluster 1 vs. cluster 2; hazard ratio (HR): 0.3, 95% confidence interval (CI): 0.1−0.7, *p* = 0.006]. Additionally, the cluster was also a statistically significant independent prognostic factor (cluster 1 vs. cluster 2; HR: 0.2, 95% CI: 0.1−0.6, *p* = 0.003) after controlling for age, sex, extent of resection, and adjuvant radiotherapy. In a similar manner as in a previous study, the extent of resection was not a significant factor for the outcomes (14).

**Table 2:** Multivariable Cox analysis of the methylation cluster and clinical features.

Covariate	Univariable Cox		Multivariable Cox	
	HR (95% CI)	*p*-value	HR (95% CI)	*p*-value
Methylation cluster (cluster 1 vs. cluster 2)	0.3 (0.1–0.7)	0.006	0.2 (0.1–0.6)	0.003
Gender (male vs. female)	1.2 (0.5–2.5)	0.703	1.2 (0.5–2.6)	0.655
Age (years)	1.0 (0.9–1.0)	0.667	1.0 (0.9–1.0)	0.285
Extent of surgery (gross total vs. subtotal)	1.6 (0.7–3.7)	0.297	0.9 (0.4–2.5)	0.911
Adjuvant radiotherapy (not received vs. received)	1.6 (0.7–3.6)	0.585	2.1 (0.8–5.2)	0.123

HR, hazard ratios; CI, confidence intervals.

A volcano plot of the probes showed that the differentially methylated sites were those with a mean β value difference (Δβ) of >0.15 and a Benjamini and Hochberg *p*-value of<0.05 (**Figure 1D**). GSEA showed that the early and late estrogen response pathways and the hypoxia pathway were activated, whereas the inflammatory and interferon gamma responses were suppressed in cluster 1 (**Figure 1E**). In addition, 46 potential therapeutic targets corresponding to differentially methylated sites were found in chordoma patients (**Supplementary Table S2**).

### Construction of the prognostic signature

**Figure 2:**
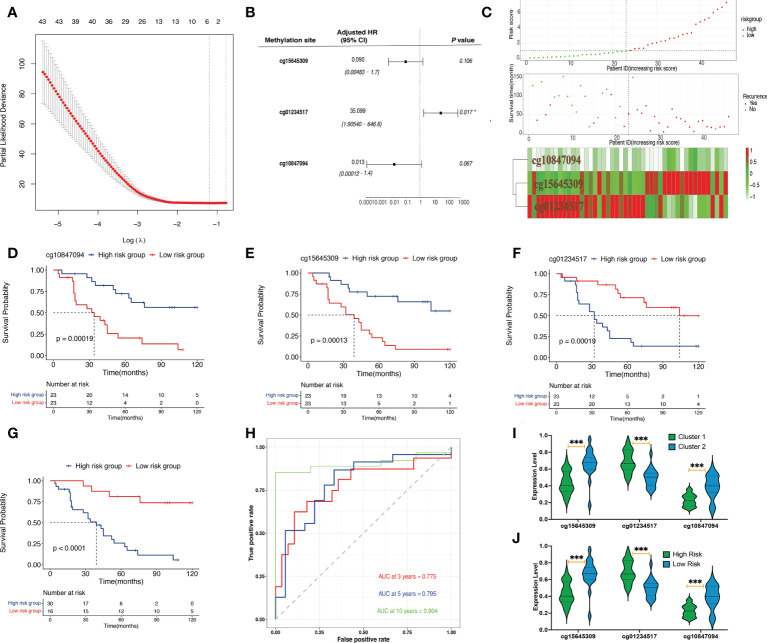
Identification and validation of prognostic signatures. **(A)** The LASSO process of developing a prognostic signature from 502 methylation sites. **(B)** The hazard ratio and the coefficients of the three methylation sites in the model calculated from multivariate Cox regression analysis. **(C)** The distribution of risk scores of 46 samples, the survival status of the 46 samples, and the heat map of the three methylation sites. **(D)** A Kaplan–Meier plot showing that the high-risk group had a significantly worse disease-specific survival time than the low-risk group. **(E–G)** A Kaplan–Meier plot showing that the high cg15645309 and cg10847094 groups had significantly worse disease-specific survival times than the low-expression group whereas the low cg01234517 group had a significantly worse disease-specific survival time than the low-expression group. **(H)** Prognostic signature analysis showing the 3-, 5-, and 10-year area-under-the-curve values of 0.775, 0.795, and 0.904, respectively. **(I)** Violin plots of the beta values of the three methylation sites between cluster 1 and cluster 2 samples. **(J)** Violin plots of the beta values of the three methylation sites between high-risk and low-risk samples (^***^ *p*< 0.001, ^*^ *p*< 0.05).

Univariate Cox regression analysis determined 502 candidate methylation sites with prognostic significance (*p*< 0.01). Of the 502 methylation sites, 5 were determined using the LASSO regression analysis (**Figure 2A**). Then, a stepwise multivariate Cox regression analysis of the five sites revealed three methylation sites (cg15645309, cg01234517, and cg10847094; **Figure 2B**). The risk score formula was as follows:


Risk score = −2.40984 × β value of cg15645309 + 3.55817 × β value of cg01234517 −−4.36864 ×β value of cg10847094

The DNA methylation level of cg01234517 was correlated with high risk, whereas cg15645309 and cg10847094 were correlated with low risk. Basic leucine zipper ATF-like transcription factor (BATF), actin-related protein 3C (ACTR3C), and fibroblast growth factor-binding protein 2 (FGFBP2) were three protein-coding genes that corresponded to the three methylation sites (cg15645309, cg01234517, and cg10847094). The results of the three methylation sites identifications, their chromosomal locations, gene types, gene symbols, feature types, coefficients, and *p-*values obtained using multivariate Cox regression analysis are listed in **Table 3**.

**Table 3:** Three cluster-related methylation sites in the prognostic signature analysis.

Probe ID	Chromosomal location	Gene type	Gene symbol	Feature type	Coefficient^a^	P value^a^
cg15645309	Chr14	Protein coding	BATF	TSS200	−2.40984	0.1061
cg01234517	Chr7:150019950–150020752	Protein coding	ACTR3C	TSS1500	3.55817	0.0167
cg10847094	Chr4	Protein coding	FGFBP2	TSS1500	−4.36864	0.0671

a In multivariate Cox regression analysis.

### Evaluating risk stratification

The patients were divided into high-risk (n = 30) and low-risk (n = 16) groups. The risk score, survival status, and heat map of the three methylation sites are shown in **Figure 2C**. The three methylation sites were associated with the outcomes of patients (*p*< 0.01; **Figures 2D–F**). In addition, low-risk patients exhibited a better DFS when compared with high-risk patients (*p*< 0.001; **Figure 2G**). The 10-, 5-, and 3-year AUCs of the predictive model were 0.904, 0.795, and 0.775, respectively (**Figure 2H**). Moreover, we found that the high-risk group and cluster 1 patients had higher methylation levels of cg01234517 and lower methylation levels of the other two sites when compared with the corresponding group (**Figures 2I, J**; *p*< 0.001).

### Immune characteristic of chordoma patients within different groups

**Figure 3:**
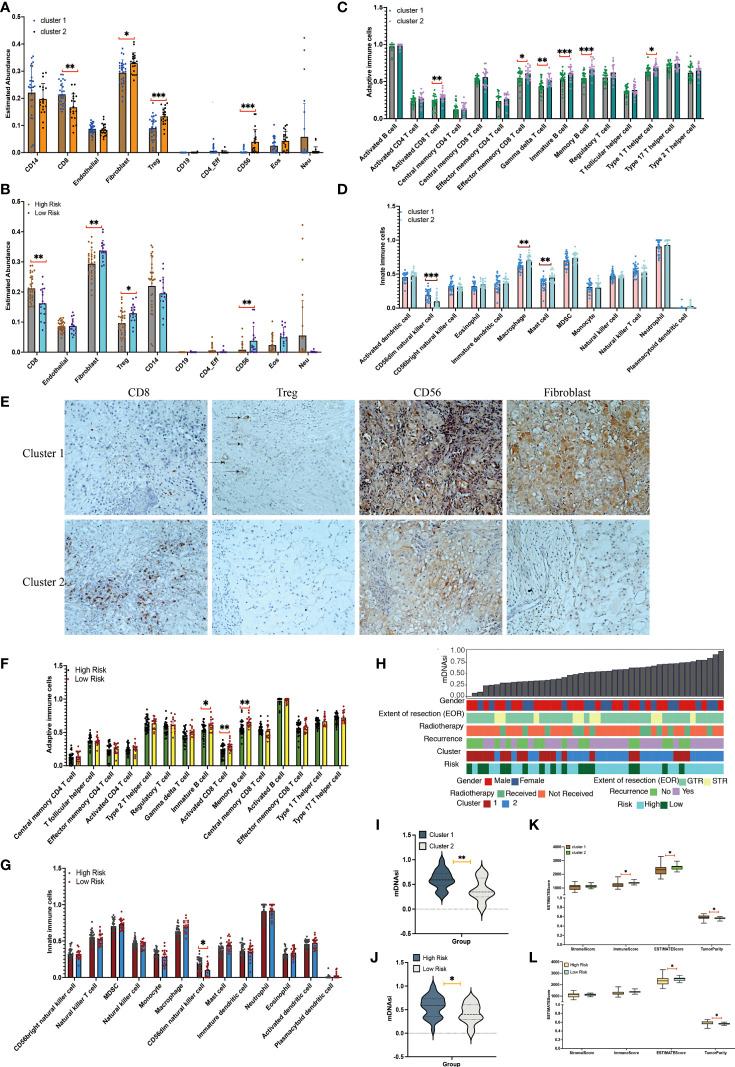
Immune cell infiltration and tumor microenvironment analyses between clusters. **(A)** Immune cell infiltration scores in cluster 1 and cluster 2 chordomas. **(B)** Immune cell infiltration scores in high-risk and low-risk chordomas. **(C)** Adaptive immune cells in cluster 1 and cluster 2 chordomas. **(D)** Innate immune cells in cluster 1 and cluster 2 chordomas. **(E)** Representative images (×20) showing more frequent presence of CD8, Treg, CD56, and fibroblasts in cluster 1 (upper panels) than cluster 2 (lower panels). **(F)** Adaptive immune cells in high-risk and low-risk chordomas. **(G)** Innate immune cells in high-risk and low-risk chordomas. **(H)** The association between known clinical and molecular features (sex, extent of resection [EOR], radiotherapy, recurrence status, cluster status, and risk status) and mDNAsi values of our cohort. Columns represent the sorted chordoma samples by mDNAsi values (low to high). Rows represent clinical information. **(I)** Violin plots of mDNAsi values in cluster 1 and cluster 2 chordoma patients. **(J)** Violin plots of mDNAsi values in the high-risk and low-risk chordoma patients. **(K)** Distribution of stromal scores, immune scores, ESTIMATE scores, and tumor purity of cluster 1 and cluster 2 chordoma patients. **(L)** Distribution of stromal scores, immune scores, ESTIMATE scores, and tumor purity of high-risk and low-risk chordoma patients (^***^ *p*< 0.001, ^**^ *p*< 0.01, ^*^ *p*< 0.05).

The methylCIBERSORT results are shown in **Figures 3A, B**. **Figure 3A** shows a greater abundance of fibroblasts (*p<* 0.05), Treg (*p*< 0.001), and CD56 (*p*< 0.001) and a lower abundance of CD8 (*p*< 0.05) in cluster 2 chordomas, which was confirmed using immunohistochemistry (**Figure 3E**). The risk group had an identical result with the cluster group. However, the low-risk group had a greater abundance of fibroblasts (*p*< 0.01), Treg (*p*< 0.05), and CD56 (*p*< 0.01) and a lower abundance of CD8 (*p*< 0.05) when compared with those of the high-risk group (**Figure 3B**).

Among the 28 immune cell types, cluster 2 was characterized by relatively high infiltration of adaptive immune and innate immune cells, including activated CD8 T cells (*p*< 0.01), effector memory CD8 T cells (*p*< 0.05), gamma delta T cells (*p*< 0.01), immature B cells (*p*< 0.001), memory B cells (*p*< 0.001), type 1 T helper cells (*p<* 0.05), macrophages (*p*< 0.01), and mast cells (*p*< 0.01; **Figures 3C, D**). In addition, cluster 2 had low infiltration of CD56dim natural killer cells (*p*< 0.001).

Additionally, the low-risk group had significantly higher proportions of immature B cells (*p*< 0.05), activated CD8 T cells (*p*< 0.01), and memory B cells (*p*< 0.01) when compared with those of the high-risk group (*p*< 0.01; **Figure 3F**). Among the 11 innate immune cell types, the high-risk group possessed significantly higher proportions of CD56dim natural killer cells when compared with those of the low-risk group (*p*< 0.05; **Figure 3G**).

Similar with the above results, analyses of tumor microenvironments also showed that cluster 1 had a lower ESTIMATE score (*p*< 0.05) and immune score (*p*< 0.05) and a higher tumor purity (*p*< 0.05) when compared with cluster 2 (**Figure 3K**). Additionally, the high-risk group exhibited a lower ESTIMATE score (*p*< 0.05) and a higher tumor purity (*p*< 0.05) when compared with the low-risk group (**Figure 3L**). Together, these results indicated that the groups had different outcomes and different immune types, and the group having better outcomes was characterized by a less immune infiltration level and activation.

### Analysis of stemness indices

The 46 chordoma samples were ranked according to the DNA methylation–based stemness index (mDNAsi) value from low to high and according to their corresponding clinical information (**Figure 3H**). The mDNAsi was assessed for the cluster and high-risk/low-risk groups. Cluster 1 and high-risk samples had higher mDNAsi values than cluster 2 (*p*< 0.01) and low-risk samples (*p*< 0.05) (**Figures 3I, J**).

### Copy number alterations of the subgroups

**Figure 4:**
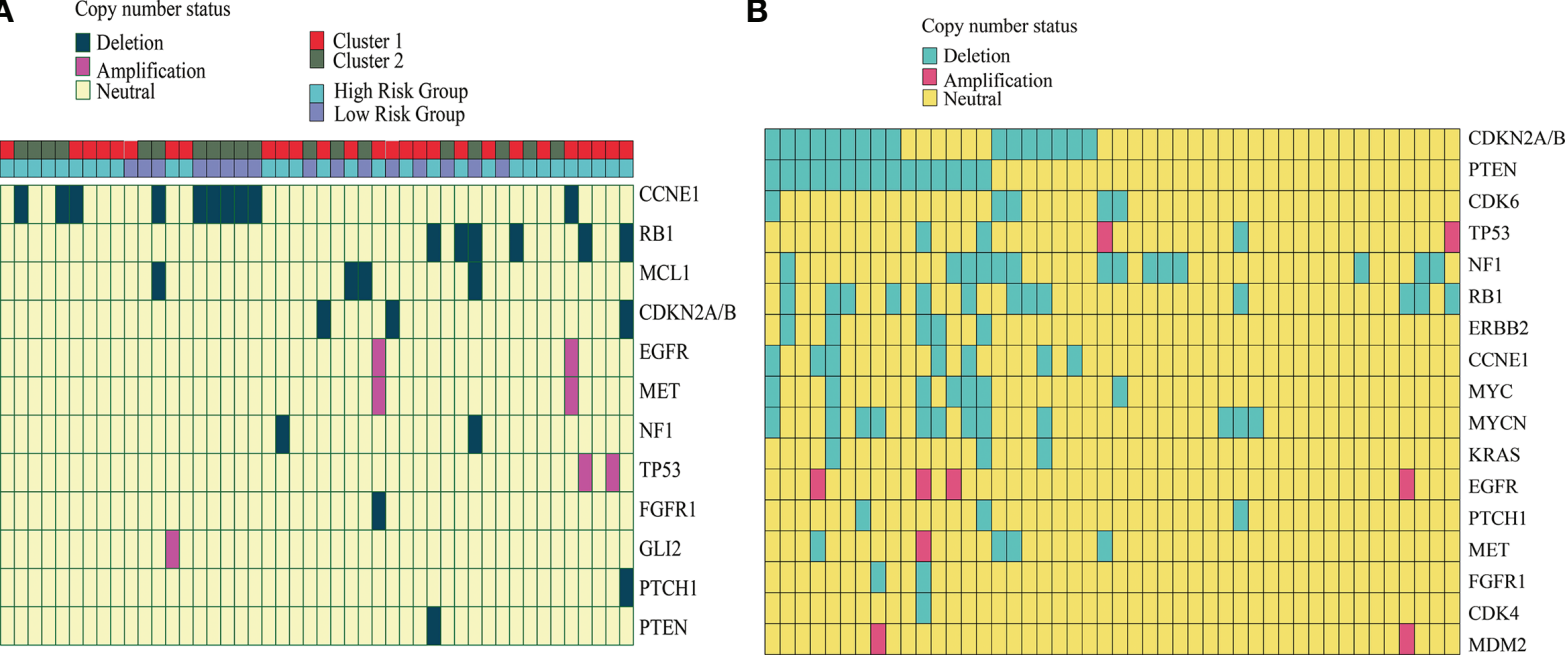
Copy number alteration of our cohort and the external GSE14068629 data set. **(A)** Copy number of the alteration plot of 46 chordoma patients and the corresponding cluster information from our cohort. **(B)** Copy number alteration plot of the 46 chordoma patients from the external GSE14068629 data set.

Copy number deletions and amplifications are shown in **Figure 4**. All amplifications occurred in cluster 1 tumors and the high-risk group, including EGFR, MET, TP53, and GLI2 (**Figure 4A**). Additionally, the presence of a CCNE1 deletion occurred exclusively in cluster 2 (8/10) chordomas and low-risk-group (6/10) chordomas, whereas all RB1 and CDKN2A/2B deletions occurred in cluster 1 and RB1 (5/6) deletions and CDKN2A/2B (2/3) deletions patients who were in the high-risk group. In the external GSE14068629 data set, the copy number alterations were identified and assessed (**Figure 4B**). CDKN2A/2B and PTEN gene deletions occurred the most, and TP53, EGFR, and MET were identified in the amplifications. CDK6, ERBB2, MYC, MYCN, KRAC, CDK4, and MDM2 were identified in the external cohort, whereas MCL1 and GLI2 were identified in our cohort.

## Discussion

Two subtypes of skull-base chordomas were identified based on DNA methylation data. The two subtypes had distinct outcomes, infiltration immune levels, activation profiles, stromal scores, tumor purities, and stemness indices. Moreover, we found that the early and late estrogen response pathways and the hypoxia pathway were activated, whereas the inflammatory and interferon gamma responses were suppressed in the worse-outcome skull-base chordomas. Previous studies reported that estrogen beta and hypoxia-inducible factor-1α were identified in chordomas (33, 34). However, their potential mechanisms of action were unclear. To further confirm that subtypes with distinct outcomes had distinctly corresponding characteristics, we divided the samples into high-risk and low-risk groups by constructing a prognostic model. In a similar manner as the clusters, high-risk and low-risk groups displayed similar outcomes, infiltration levels, activations, stemness indices, and tumor purities.

The types of treatments for skull-base chordomas may result from tumor heterogeneity, which potentially responds to different treatments. These subtypes displayed skull-base chordoma subtypes that may reveal heterogeneity despite their corresponding clinical features. The information could contribute to resolving chordoma outcomes that are clinically observed despite standards-of-care treatments. In addition, subtypes with a better DFS had higher fractions of immune cells within tumors, which included fibroblasts, Tregs, and CD56, whereas the subtype with better DFS had higher fractions of CD56dim natural killer cells. Similar with our results, some studies reported that chordomas with distinct outcomes had distinct immune levels, and chordoma patients with better DFS had higher fractions of immune cells (14, 35, 36). Additionally, our recently published study has revealed that inflammatory activity–associated proteins were associated with the invasion characteristics of skull-base chordoma (37). As for stemness indices, a study showed that stem cell signature expressions correlated inversely with patient survival (38, 39). In a similar manner with this report, the worse DFS subgroups exhibited higher expression levels of stem cell genes when compared with the contrast group. The protein coding genes corresponding to the three methylation sites in the prognostic model were BATF, ACTR3C, and FGFBP2. Additionally, BATF, ACTR3C, and FGFBP2 have been implicated in cancers other than chordomas (40–42). However, the functional effects of the three aberrant methylation genes require more studies to elucidate. Notably, BATF-driven gene regulation could be used as a potential target for delaying CD8 T-cell aging and restoring function (43). Importantly, some studies have reported that BATF was correlated with the response of immune cells in humans (44–46). ACTR3C was found hypermethylated in keloids, and the mRNA expression of ACTR3C was statistically significantly different between keloid and normal skin (47, 48). Moreover, CD8^+^ FGFBP2^+^ T cells and FGFBP2^+^ natural killer cells were found to display high levels of cytotoxic effectors and low levels of inhibitory markers (49). FGFBP2 showed six- to eightfold higher levels in clear cell stage I carcinomas compared with the more advanced staged carcinomas and correlated positively with an improved clinical outcome (42). The molecular differences may help these subtypes to respond separately to specific therapeutic approaches.

Finally, all amplifications occurring in subgroups had worse outcomes. The presence of a CCNE1 deletion was seen exclusively in patients with better outcome, whereas all RB1 and CDKN2A/2B deletions occurred in cluster 1. Similarly, CCNE1 deletions in gliomas were also correlated with a better outcome (50), which was similar with our result. In another study, the authors also revealed that CDKN2A/2B deletions were found in skull-base chordoma patients (51), but the underlying mechanism was unknown. RB1 and TrP53 could cooperate to suppress prostate cancer lineage plasticity, metastasis, and antiandrogen resistance (52).

This study described methylation-based reclassification and risk stratification of skull-base chordomas. However, the sample size was limited because of the relative rarity of chordomas. Whether the immune infiltration level and activation, along with copy number variation and stemness indices, could be used as prognostic features or potential therapeutic targets in skull-base chordomas needs to be studied in the future. Additionally, the subtypes should be validated in another independent data set to assess their prognostic performance.

## Data availability statement

The raw data were deposited in the GEO data set with GEO number GSE205331. Requests for clinical information or information of any data provided in the study can be directed to hxl950513@ccmu.edu.cn.

## Ethics statement

The studies involving human participants were reviewed and approved by Beijing Tiantan Hospital. The patients/participants provided their written informed consent to participate in this study. The animal study was reviewed and approved by Beijing Tiantan Hospital. Written informed consent was obtained from the individual(s) for the publication of any potentially identifiable images or data included in this article.

## Author contributions

Conceptualization, ZW and LW; methodology, XH and WC; software, XH and TG; validation, BY, DL, and HL; formal analysis, XH; investigation, DL; resources, DL; data curation, TG; writing—original draft preparation, XH, TG, and WC; writing—review and editing, XH, TG, and WC; visualization, XH; supervision, ZW and LW; project administration, KW; funding acquisition, KW. All authors have read and agreed to the published version of the manuscript. All authors contributed to the article and approved the submitted version.

## Funding

This work wa**s** supported by the National Natural Science Foundation of China (Nos. 62027813, 2022YFE0112500, and 8180100922), the Beijing Municipal Science and Technology Commission (No. 7192056), and the Natural Science Foundation of Beijing (No. J180005).

## Conflict of interest

The authors declare that the research was conducted in the absence of any commercial or financial relationships that could be construed as a potential conflict of interest.

## Publisher’s note

All claims expressed in this article are solely those of the authors and do not necessarily represent those of their affiliated organizations, or those of the publisher, the editors and the reviewers. Any product that may be evaluated in this article, or claim that may be made by its manufacturer, is not guaranteed or endorsed by the publisher.

## Supplementary material

Supplementary Table 1 Clinical information of 46 patients.

Supplementary Table 2 Relationship between potential drugs with chordomas (the Open Targets platform).

## References

[1] Salisbury JR Deverell MH Cookson MJ Whimster WF. Three-dimensional reconstruction of human embryonic notochords: clue to the pathogenesis of chordoma. J Pathol (1993) 171(1):59–62. doi: 10.1002/path.1711710112 8229458

[2] Boriani S Bandiera S Biagini R Bacchini P Boriani L Cappuccio M. Chordoma of the mobile spine: fifty years of experience. Spine (Phila Pa 1976) (2006) 31(4):493–503. doi: 10.1097/01.brs.0000200038.30869.27 16481964

[3] Jones PS Aghi.MK Muzikansky A Shih HA Barker FG 2nd Curry WT. Outcomes and patterns of care in adult skull base chordomas from the surveillance, epidemiology, and end results (SEER) database. J Clin Neurosci (2014) 21(9):1490–6. doi: 10.1016/j.jocn.2014.02.008 24852903

[4] Neri F Rapelli S Krepelova A Incarnato D Parlato C Basile G. Intragenic DNA methylation prevents spurious transcription initiation. Nature (2017) p:72–7. doi: 10.1038/nature21373 28225755

[5] Noushmehr H Weisenberger DJ Diefes K Phillips HS Pujara K Berman BP. Identification of a CpG island methylator phenotype that defines a distinct subgroup of glioma. Cancer Cell (2010) 17(5):510–22. doi: 10.1016/j.ccr.2010.03.017 PMC2872684 20399149

[6] Brennan K Koenig JL Gentles AJ Sunwoo JB Gevaert O. Identification of an atypical etiological head and neck squamous carcinoma subtype featuring the CpG island methylator phenotype. EBioMedicine (2017) 17:223–36. doi: 10.1016/j.ebiom.2017.02.025 PMC5360591 28314692

[7] Barreau O Assie G Wilmot-Roussel H Ragazzon B Baudry C Perlemoine K. Identification of a CpG island methylator phenotype in adrenocortical carcinomas. J Clin Endocrinol Metab (2013) 98(1):E174–84. doi: 10.1210/jc.2012-2993 23093492

[8] Guo W Zhu L Zhu R Chen Q Wang Q Chen JQ. A four-DNA methylation biomarker is a superior predictor of survival of patients with cutaneous melanoma. Elife (2019) 8:e44310. doi: 10.7554/eLife.44310 31169496 PMC6553943

[9] Guo W Zhu L Yu M Zhu R Chen Q Wang Q. A five-DNA methylation signature act as a novel prognostic biomarker in patients with ovarian serous cystadenocarcinoma. Clin Epigenet (2018) 10(1):142. doi: 10.1186/s13148-018-0574-0 PMC6240326 30446011

[10] Tao C Luo R Song J Zhang W Ran L. A seven-DNA methylation signature as a novel prognostic biomarker in breast cancer. J Cell Biochem (2020) 121(3):2385–93. doi: 10.1002/jcb.29461 31646666

[11] Capper D Jones DTW Sill M Hovestadt V Schrimpf D Sturm D. DNA Methylation-based classification of central nervous system tumours. Nature (2018) 555(7697):469–74. doi: 10.1038/nature26000 PMC6093218 29539639

[12] Alholle A Brini AT Bauer J Gharanei S Niada S Slater A. Genome-wide DNA methylation profiling of recurrent and non-recurrent chordomas. Epigenetics (2015) 10(3):213–20. doi: 10.1080/15592294.2015.1006497 PMC4622541 25621392

[13] Rinner B Weinhaeusel A Lohberger B Froehlich EV Pulverer W Fischer C. Chordoma characterization of significant changes of the DNA methylation pattern. PloS One (2013) 8(3):e56609. doi: 10.1371/journal.pone.0056609 23533570 PMC3606365

[14] Zuccato JA Patil V Mansouri S Liu JC Nassiri F Mamatjan Y. DNA Methylation based prognostic subtypes of chordoma tumors in tissue and plasma. Neuro Oncol (2021) 24(3):442–54. doi: 10.1093/neuonc/noab235 PMC8917394 34614192

[15] Meng T Huang R Jin J Gao J Liu F Wei Z. A comparative integrated multi-omics analysis identifies CA2 as a novel target for chordoma. Neuro Oncol (2021) 23(10):1709–22. doi: 10.1093/neuonc/noab156 PMC8485454 34214167

[16] Cottone L Cribbs AP Khandelwal G Wells G Ligammari L Philpott M. Inhibition of histone H3K27 demethylases inactivates brachyury (TBXT) and promotes chordoma cell death. Cancer Res (2020) 80(20):4540–51. doi: 10.1158/0008-5472.CAN-20-1387 PMC7616956 32855205

[17] Wilkerson MD Hayes DN. ConsensusClusterPlus: A class discovery tool with confidence assessments and item tracking. Bioinformatics (2010) 26(12):1572–3. doi: 10.1093/bioinformatics/btq170 PMC2881355 20427518

[18] Dietz S Lifshitz A Kazdal D Harms A Endris V Winter H. Global DNA methylation reflects spatial heterogeneity and molecular evolution of lung adenocarcinomas. Int J Cancer (2019) 144(5):1061–72. doi: 10.1002/ijc.31939 30350867

[19] Subramanian A Tamayo P Mootha VK Mukherjee S Ebert BL Gillette MA. Gene set enrichment analysis: A knowledge-based approach for interpreting genome-wide expression profiles. Proc Natl Acad Sci U.S.A. (2005) 102(43):15545–50. doi: 10.1073/pnas.0506580102 PMC1239896 16199517

[20] Dong D Tian Y Zheng SC Teschendorffeb AE. ebGSEA: an improved gene set enrichment analysis method for epigenome-Wide-Association studies. Bioinformatics (2019) 35(18):3514–6. doi: 10.1093/bioinformatics/btz073 PMC6748733 30715212

[21] Ochoa D Hercules A Carmona M Suveges D Gonzalez-Uriarte A Malangone C. Open targets platform: supporting systematic drug-target identification and prioritisation. Nucleic Acids Res (2021) 49(D1):D1302–10. doi: 10.1093/nar/gkaa1027 PMC7779013 33196847

[22] Wang H Lengerich BJ Aragam B Xing EP. Precision lasso: accounting for correlations and linear dependencies in high-dimensional genomic data. Bioinformatics (2019) 35(7):1181–7. doi: 10.1093/bioinformatics/bty750 PMC6449749 30184048

[23] Lorent M Giral M Foucher Y. Net time-dependent ROC curves: a solution for evaluating the accuracy of a marker to predict disease-related mortality. Stat Med (2014) 33(14):2379–89. doi: 10.1002/sim.6079 24399671

[24] Charoentong P Finotello F Angelova M Mayer C Efremova M Rieder D. Pan-cancer immunogenomic analyses reveal genotype-immunophenotype relationships and predictors of response to checkpoint blockade. Cell Rep (2017) 18(1):248–62. doi: 10.1016/j.celrep.2016.12.019 28052254

[25] Song LR Weng JC Li CB Huo XL Li H Hao SY. Prognostic and predictive value of an immune infiltration signature in diffuse lower-grade gliomas. JCI Insight (2020) 5(8):e133811. doi: 10.1172/jci.insight.133811 32229719 PMC7205440

[26] Baghban R Roshangar L Jahanban-Esfahlan R Seidi K Ebrahimi-Kalan A Jaymand M. Tumor microenvironment complexity and therapeutic implications at a glance. Cell Commun Signal (2020) 18(1):59. doi: 10.1186/s12964-020-0530-4 32264958 PMC7140346

[27] Newman AM Liu CL Green MR Gentles AJ Feng W Xu Y. Robust enumeration of cell subsets from tissue expression profiles. Nat Methods (2015) 12(5):453–7. doi: 10.1038/nmeth.3337 PMC4739640 25822800

[28] Yoshihara K Shahmoradgoli M Martinez E Vegesna R Kim H Torres-Garcia W. Inferring tumour purity and stromal and immune cell admixture from expression data. Nat Commun (2013) 4:2612. doi: 10.1038/ncomms3612 24113773 PMC3826632

[29] Daily K Ho Sui SJ Schriml LM Dexheimer PJ Salomonis N Schroll R. Molecular, phenotypic, and sample-associated data to describe pluripotent stem cell lines and derivatives. Sci Data (2017) 4:170030. doi: 10.1038/sdata.2017.30 28350385 PMC5369318

[30] Salomonis N Dexheimer PJ Omberg L Schroll R Bush S Huo J. Integrated genomic analysis of diverse induced pluripotent stem cells from the progenitor cell biology consortium. Stem Cell Rep (2016) 7(1):110–25. doi: 10.1016/j.stemcr.2016.05.006 PMC4944587 27293150

[31] Capper D Stichel D Sahm F Jones DTW Schrimpf D Sill M. Practical implementation of DNA methylation and copy-number-based CNS tumor diagnostics: the Heidelberg experience. Acta Neuropathol (2018) 136(2):181–210. doi: 10.1007/s00401-018-1879-y 29967940 PMC6060790

[32] Koelsche C Schrimpf D Stichel D Sill M Sahm F Reuss DE. Sarcoma classification by DNA methylation profiling. Nat Commun (2021) 12(1):498. doi: 10.1038/s41467-020-20603-4 33479225 PMC7819999

[33] Li X Ji Z Ma Y Qiu X Fan Q Ma B. Expression of hypoxia-inducible factor-1α, vascular endothelial growth factor and matrix metalloproteinase-2 in sacral chordomas. Oncol Lett (2012) 3(6):1268–74. doi: 10.3892/ol.2012.645 PMC3392560 22783431

[34] Fasig JH Dupont WD Olson SJ Lafleur BJ Cates JM. Steroid hormone receptor and COX-2 expression in chordoma. Am J Clin Pathol (2007) 128(3):375–81. doi: 10.1309/8T2NPHLK5X5WQ3E7 17709310

[35] Hu W Yu J Huang Y Hu F Zhang X Wang Y. Lymphocyte-related inflammation and immune-based scores predict prognosis of chordoma patients after radical resection. Transl Oncol (2018) 11(2):444–9. doi: 10.1016/j.tranon.2018.01.010 PMC5842326 29477108

[36] Li M Bai J Wang S Zhai Y Zhang S Li C. Clinical implication of systemic immune-inflammation index and prognostic nutritional index in skull base chordoma patients. Front Oncol (2021) 11:548325. doi: 10.3389/fonc.2021.548325 33718126 PMC7947628

[37] Wu Z Wang L Guo Z Wang K Zhang Y Tian K. Experimental study on differences in clivus chordoma bone invasion: an iTRAQ-based quantitative proteomic analysis. PloS One (2015) 10(3):e0119523. doi: 10.1371/journal.pone.0119523 25793716 PMC4368785

[38] Lian H Han YP Zhang YC Zhao Y Yan S Li QF. Integrative analysis of gene expression and DNA methylation through one-class logistic regression machine learning identifies stemness features in medulloblastoma. Mol Oncol (2019) 13(10):2227–45. doi: 10.1002/1878-0261.12557 PMC6763787 31385424

[39] Zheng X Naiditch J Czurylo M Jie C Lautz T Clark S. Differential effect of long-term drug selection with doxorubicin and vorinostat on neuroblastoma cells with cancer stem cell characteristics. Cell Death Dis (2013) 4:e740. doi: 10.1038/cddis.2013.264 23887631 PMC3730434

[40] Schleussner N Merkel O Costanza M Liang HC Hummel F Romagnani C. The AP-1-BATF and -BATF3 module is essential for growth, survival and TH17/ILC3 skewing of anaplastic large cell lymphoma. Leukemia (2018) 32(9):1994–2007. doi: 10.1038/s41375-018-0045-9 29588546 PMC6127090

[41] Topchyan P Xin G Chen Y Zheng S Burns R Shen J. Harnessing the IL-21-BATF pathway in the CD8(+) T cell anti-tumor response. Cancers (Basel) (2021) 13(6):1263. doi: 10.3390/cancers13061263 33809259 PMC7998696

[42] Elgaaen BV Haug KB Wang J Olstad OK Fortunati D Onsrud M. POLD2 and KSP37 (FGFBP2) correlate strongly with histology, stage and outcome in ovarian carcinomas. PloS One (2010) 5(11):e13837. doi: 10.1371/journal.pone.0013837 21079801 PMC2973954

[43] Moskowitz DM Zhang DW Hu B Saux Le S Yanes RE Ye Z. Epigenomics of human CD8 T cell differentiation and aging. Sci Immunol (2017) 2(8):eaag0192. doi: 10.1126/sciimmunol.aag0192 28439570 PMC5399889

[44] Liu Q Kim MH Friesen L Kim CH. BATF regulates innate lymphoid cell hematopoiesis and homeostasis. Sci Immunol (2020) 5(54):peaaz8154. doi: 10.1126/sciimmunol.aaz8154 PMC8375455 33277375

[45] Glasmacher E Agrawal S Chang AB Murphy TL Zeng W Vander Lugt B. A genomic regulatory element that directs assembly and function of immune-specific AP-1-IRF complexes. Science (2012) 338(6109):975–80. doi: 10.1126/science.1228309 PMC5789805 22983707

[46] Li P Spolski R Liao W Wang L Murphy TL Murphy KM. BATF-JUN is critical for IRF4-mediated transcription in T cells. Nature (2012) 490(7421):543–6. doi: 10.1038/nature11530 PMC3537508 22992523

[47] Garcia-Rodriguez L Jones L Chen KM Datta I Divine G Worsham MJ. Causal network analysis of head and neck keloid tissue identifies potential master regulators. Laryngoscope (2016) 126(10):E319–24. doi: 10.1002/lary.25958 26990118

[48] Jones LR Greene J Chen KM Divine G Chitale D Shah V. Biological significance of genome-wide DNA methylation profiles in keloids. Laryngoscope (2017) 127(1):70–8. doi: 10.1002/lary.26063 27312686

[49] Li X Zhang M Lei T Zou W Huang R Wang F. Single-cell RNA-sequencing dissects cellular heterogeneity and identifies two tumor-suppressing immune cell subclusters in HPV-related cervical adenosquamous carcinoma. J Med Virol (2022) 94(12):6047–59. doi: 10.1002/jmv.28084 36000446

[50] Vogazianou AP Chan R Backlund LM Pearson DM Liu L Langford CF. Distinct patterns of 1p and 19q alterations identify subtypes of human gliomas that have different prognoses. Neuro Oncol (2010) 12(7):664–78. doi: 10.1093/neuonc/nop075 PMC2940668 20164239

[51] Bai J Shi J Li C Wang S Zhang T Hua X. Whole genome sequencing of skull-base chordoma reveals genomic alterations associated with recurrence and chordoma-specific survival. Nat Commun (2021) 12(1):757. doi: 10.1038/s41467-021-21026-5 33536423 PMC7859411

[52] Ku SY Rosario S Wang Y Mu P Seshadri M Goodrich ZW. Rb1 and Trp53 cooperate to suppress prostate cancer lineage plasticity, metastasis, and antiandrogen resistance. Science (2017) 355(6320):78–83. doi: 10.1126/science.aah4199 28059767 PMC5367887

